# Severe Hypoglycaemia in an Elderly Patient With Alzheimer's Dementia and Type 2 Diabetes Mellitus: A Case Report

**DOI:** 10.7759/cureus.49423

**Published:** 2023-11-26

**Authors:** Amit Banerjee, Moe Wutye Kyaw

**Affiliations:** 1 Diabetes and Endocrinology, Royal Liverpool Hospital, Liverpool, GBR; 2 Internal Medicine, St Helens and Knowsley NHS Foundation Trust, Barrow-in-Furness, GBR

**Keywords:** dextrose, octreotide, sulphonylurea oral hypoglycaemic, sulfonylurea overdose, hypoglycaemia

## Abstract

A 78-year-old diabetic patient was admitted with unexplained refractory hypoglycaemia. He was taking gliclazide and metformin and had good glycaemic control. He was suspected to have a sulfonylurea overdose, and intravenous 10% dextrose failed to correct hypoglycaemia. He was then treated with octreotide (a specific antidote to sulfonylurea), which corrected his low blood sugar level. Intravenous dextrose and octreotide are often the drugs of choice for treating hypoglycaemia from sulfonylurea toxicity. A high index of suspicion is needed for early diagnosis of sulfonylurea overdose.

## Introduction

This case report presents a challenging clinical scenario of an elderly 78-year-old male with early-stage Alzheimer's dementia and type 2 diabetes mellitus (T2DM) who experienced severe hypoglycaemia, likely resulting from an accidental overdose of gliclazide, a sulfonylurea medication commonly used to manage diabetes. The sulfonylurea group of medication causes the closure of adenosine triphosphate (ATP)-sensitive K-channels in the beta-cell plasma membrane, initiating a chain of events, which results in insulin release [1]. The most common side effects of the sulfonylurea group of medications are hypoglycaemia, weight gain, nausea, vomiting, and loose motion [2].

## Case presentation

The patient was on a stable regimen of metformin 1 gm twice daily and gliclazide 40 mg once daily for glycaemic control, with a recent glycosylated haemoglobin level of 52 mmol/mol. He has been on these diabetic medications for seven years now. His diabetes is reasonably well controlled.

His wife called an ambulance service when she found him drowsy at lunchtime. Upon evaluation from the ambulance crew, the patient's capillary blood glucose (CBG) was critically low at 2.1 mmol/L (37.8 mg/dL). This was corrected by the ambulance crew with a bolus of 250 ml of 10% dextrose intravenously. He was brought to the emergency department and suffered multiple hypoglycaemic episodes in the resuscitation room, needing glucagon injection and IV dextrose infusion. He was initially started on a 5% dextrose infusion at 125 ml/ hour and then a 10% dextrose infusion at 100 ml/hour. But he kept on having hypoglycaemic episodes (blood glucose level < 3.5 mmol/L).

The patient took his morning medication (two 500 mg tablets of metformin and one 40 mg tablet of gliclazide) himself despite his dementia, and his wife could not provide any information regarding any missing medication or accidental overdose. The patient denied any overdose. However, he acknowledged that his memory has deteriorated in the last two years due to the progression of his dementia, making him more forgetful.

The patient's blood pressure and pulse were 136/76 mmHg and 88 bpm, respectively. His random cortisol, thyroid function, lactic acid, and electrolyte levels were normal. His renal function and full blood count were also normal. His venous blood gas revealed a pH of 7.41 (which was within the normal range) (Tables 1, 2).

**Table 1:** Vital parameters of the patient.

Vital observations	
Blood pressure	136/76 mm Hg
Heart rate	88 beats per minute
Respiratory rate	14 breaths per minute
Oxygen saturation	96% on room air
Body temperature (axillary)	36.8°C

**Table 2:** Laboratory results of the patient.

	Results	Normal range
Full blood count		
Haemoglobin	13.6 g/dL	13.8-17.2 g/dL
WBC	8,000 cells/mcL	4,500-11,000 cells/mcL
Urea and electrolytes		
Sodium (Na)	137 mmol/L	135-145 mmol/L
Potassium (K)	4.7 mmol/L	3.5-5.1 mmol/L
Urea	7.7 mg/dL	7-20 mg/dL
Creatinine	0.7 mg/dL	0.6-1.2 mg/dL
Bicarbonate (HCO3)	26 mmol/L	22-28 mmol/L
Random cortisol	17.4 mcg/dL	6.2-19.4 mcg/dL
Thyroid function test		
Thyroid-stimulating hormone	2.3 mIU/L	0.4-4.0 mIU/L
Free T4	1.4 ng/dL	0.8-1.8 ng/dL
Lactic acid	2.3 mmol/L	0.5-2.2 mmol/L
pH (in venous blood sample)	7.41	
Plasma paracetamol level	<10 mg/L	
Random venous blood glucose	3.8 mmol/L	3.9-7.8 mmol/L

Clinicians strongly suspected an accidental overdose of sulfonylurea. His urine sample was sent for a sulfonylurea level test. The sulfonylurea level was reported from the lab as positive (ug/mL). Based on the strong clinical suspicion of sulfonylurea overdose, he was treated with a stat dose of 50 mcg of octreotide subcutaneously, which effectively stabilized his blood glucose level. No further hypoglycaemic episode was recorded despite checking blood glucose levels hourly. He was kept on a 5% dextrose infusion for 24 hours and observed in hospital for 48 hours. He was discharged back to his home after 48 hours. His gliclazide medication was stopped. He will be followed up by the diabetic team.

## Discussion

This patient was having refractory hypoglycaemia. Clinical suspicion was of overdose with metformin or gliclazide or both. Metformin overdose very rarely causes hypoglycaemia. However, it can cause severe lactic acidosis (especially in patients with renal impairment), which can become life-threatening in extreme cases [1]. Such critical lactic acidosis from metformin overdose needs haemofiltration or haemodialysis and consideration for escalation to the intensive therapy unit [2-5]. However, this patient’s renal function and lactic acid level were normal. So, a significant metformin overdose was unlikely.

Sulfonylurea (gliclazide in this case) can cause profound and prolonged hypoglycaemia [1]. The onset of hypoglycaemia occurs within a few hours of ingestion and, without treatment, can progress in severity [1]. Haemodialysis or haemofiltration is ineffective in the treatment of sulfonylurea overdose. Decontamination of the gut was considered but deemed less likely to be effective. The medication was likely to be ingested with breakfast four hours before.

Dextrose is a specific antidote for sulfonylurea poisoning and is central to the resuscitation of the sulfonylurea-poisoned patient. However, attempts to maintain euglycaemia by continued infusion of concentrated dextrose are problematic, as glucose administration stimulates further insulin release and can cause rebound hypoglycaemia [6]. Administration of 10% dextrose risks phlebitis and necessitates a central venous line, which is problematic for a 78-year-old patient with dementia. That is why it is essential to consider specific antidotes.

Diazoxide is no longer advocated as a sulfonylurea antidote due to its side effect profile [7,8]. By contrast, octreotide, a synthetic octapeptide analogue of somatostatin, effectively suppresses insulin secretion from beta cells of the pancreas and has a very benign adverse effect profile [7]. It appears to abolish the need for hypertonic dextrose infusion. Octreotide is now regarded as a first-line antidote for sulfonylurea poisoning, with the role of dextrose confined to rapid restoration of euglycaemia in the already hypoglycaemic patient and maintenance of euglycaemia until octreotide can be sourced and administered [1]. Octreotide can be administered as 50 to 100 mcg IV or subcutaneously six hourly or as an infusion of 25 to 50 mcg/hour [6].

## Conclusions

Accidental overdose of gliclazide in elderly patients with cognitive impairment can lead to severe and refractory hypoglycaemia. Timely recognition, supportive care with dextrose infusion, and the use of octreotide can effectively manage such cases.
